# Segmental aneuploidy in human blastocysts: a qualitative and quantitative overview

**DOI:** 10.1186/s12958-019-0515-6

**Published:** 2019-09-16

**Authors:** María-José Escribà, Xavier Vendrell, Vanessa Peinado

**Affiliations:** 1 IVF Laboratory. IVIRMA-Valencia, 46015 Valencia, Spain; 2 Reproductive Genetics Unit, Sistemas Genómicos, Parque Tecnológico Paterna, 46980 Valencia, Spain; 3 Igenomix, Parque Tecnológico Paterna, 46980 Valencia, Spain

**Keywords:** Human blastocyst, Segmental aneuploidy, Chromosomal instability, PGT-A, Chromothripsis

## Abstract

**Background:**

Microarray-based and next generation sequencing (NGS) technologies have revealed that segmental aneuploidy is frequently present in human oocytes, cleavage-stage embryos and blastocysts. However, very little research has analyzed the type, size, chromosomal distribution and topography of the chromosomal segments at the different stages of development.

**Methods:**

This is a retrospective study of 822 PGT-A (preimplantation genetic test for aneuploidies) performed on trophectoderm samples from 3565 blastocysts biopsied between January 2016 and April 2017. The cycles in question had been initiated for varying clinical indications. Samples were analyzed by next generation sequencing-based technology. Segmental aneuploidies were evaluated when fragment size was > 5 Mb. Blastocysts presenting a single segmental aneuploidy (SSA), without any additional whole-chromosome gain/loss, were statistically analyzed for incidence, type, size and chromosomal emplacement. Segment sizes relative to the whole chromosome or arm (chromosome- and arm-ratios) were also studied.

**Results:**

8.4% (299/3565) of blastocysts exhibited segmental aneuploidy for one or more chromosomes, some of which were associated with whole-chromosome aneuploidy while others were not. Nearly half of them (4.5%: 159/3565 of blastocysts) exhibited pure-SSA, meaning that a single chromosome was affected by a SSA. Segments were more frequent in medium-sized metacentric or submetacentric chromosomes and particularly in q-chrmosome arms, variables that were related to trophectoderm quality. SSA size was related to a greater extent to chromosome number and the arm affected than it was to SSA type. In absolute values (Mb), SSA size was larger in large chromosomes. However, the SSA:chromosome ratio was constant across all chromosomes and never exceeded 50% of the chromosome.

**Conclusions:**

SSA frequency is chromosome- and topographically dependent, and its incidence is not related to clinical or embryological factors, but rather to trophectoderm quality. SSA might be originated by chromosome instability in response to chromothripsis, bias introduced by the biopsy and/or iatrogenic effects.

**Trial registration:**

Retrospectively registered.

**Electronic supplementary material:**

The online version of this article (10.1186/s12958-019-0515-6) contains supplementary material, which is available to authorized users.

## Background

### The “limits” of embryo aneuploidy detection

Certain morphologically normal euploid embryos fail to culminate in a live birth. This could be due to embryonic, endometrial or epigenetic factors or, as recently suggested, subchromosomal abnormalities or embryonic mosaicism [1]. In this paper, we focus firstly on partial subchromosomal gains and losses on the p- or q-chromosome arm – referred to as “segmental aneuploidies” - including their frequency and origin. Secondly, we describe a population of blastocysts with segmental aneuploidies, analyzed by next-generation sequencing (NGS).

Segmental aneuploidies are generated when a small piece of a chromosome is gained or lost during cell division, resulting in subchromosomal copy number (CN) changes [2, 3]. The ability to detect segmental imbalances in preimplantation embryos depends on the method used for chromosomal analysis and the limits to its power of detection (resolution). Currently, there are several platforms used for chromosomal studies of human oocytes and preimplantation embryos (reviewed by Brezina et al. [4]). Each platform varies in its measuring capacity. Some “low-resolution” preimplantation genetic tests for aneuploidy (PGT-A), such as fluorescence in situ hybridization (FISH), bacterial artificial chromosomes (BACs)-on-beads (BOBs), or quantitative real-time polymerase chain reaction (qRT-PCR), have been applied to infer whole-chromosome aneuploidy, but they are unable to accurately identify variations of intrachromosomal dosage. Other PGT-A methods, such as comparative genomic hybridization (CGH), single nucleotide polymorphism (SNP)-based microarrays (reviewed by Vanneste et al. [5]) and NGS technologies, have been used in clinical practice to detect both segmental and whole chromosomal aneuploidy.

CGH-based microarrays (aCGH) have been extensively employed in PGT-A, but they struggle to accurately detect low-rate aneuploidy (mosaicism) and segmental aneuploidy in trophectoderm (TE) samples. In this context, SNP-aCGH can detect segmental aneuploidies as small as 13.8 Mb [6] and the CN resolution of BAC-aCGH is approximately 20 Mb. However, using BAC-aCGH, some authors detected segmental aneuploidies as small as 5 Mb or 6 Mb in TE and blastomere samples, respectively [7].

Several platforms and protocols based on NGS have been validated [8–15], and can be employed to assess altered distribution patterns of segmental aneuploidies. Previous studies using NGS technology have described a minimum size of almost 14 Mb for detecting imbalances [9, 16, 17], although other authors have reported imbalances of 1.5–1.8 Mb [14, 15] and segmental aneuploidies as small as 10.0 Mb [18].

### Bias and artefacts

Most PGT-A methods require pre-treatment of whole genome amplification (WGA), which can introduce artifacts that can be misinterpreted as “true” segmental imbalances. To date, all available WGA methods have resulted in a biased representation of the original genome as a result of the allele drop-out, preferential amplification, structural DNA anomalies or nucleotide copy error [19]. A source of error are S-phase artifacts, whereby single-cell DNA replication domains can result in copy number changes that are interpreted as segmental aneuploidy [20]. In fact, Dimitriadou et al. [20] reported that detection of segmental aneuploidies in single cells is not conclusive during the S-phase of the cell cycle.

### Current questions

Segmental aneuploidies account for approximately 6% of clinical miscarriages (analyzed by FISH) [21] and close to 0.05% of newborns (analyzed by FISH and PCR) [22], which is in line with the frequency of segmental aneuploidies detected in oocytes, preimplantation embryos [23] and blastocysts [24, 25], all of which implies that this abnormality should be taken into account.

Considering this scenario, the aim of the present work was to study the incidence of segmental aneuploidies in a population of biopsied blastocysts and to relate it to certain medical indications and blastocyst quality. Additionally, we aimed to describe qualitative and quantitative types of segmental aneuploidy and to determine whether a preferential chromosomal-dependent effect exists.

## Materials and methods

### Study population and design

This retrospective study includes 3565 blastocysts pertaining to 822 cycles of PGT-A. All TE samples were obtained from blastocysts between January 2016 and April 2017 and were analyzed by NGS-based technology. The patients enrolled in the study received medical counseling and signed a consent form. The study was approved by the ethics committee of IVIRMA-Valencia, Spain (1710-VLC-101-ME).

Assisted reproduction methodologies, including hormone stimulation protocols, oocyte retrieval, in vitro fertilization by intracytoplasmic sperm injection (ICSI), extended in vitro culture, blastocyst biopsy and vitrification, were carried out according to standard protocols used at IVIRMA-Valencia [26]. In all cases, blastocyst biopsy was performed by laser-assisted methodologies on day 5 or 6 after fertilization. Cell biopsies were obtained by aspiration of the TE portion (hernia), which contains approximately five cells.

### Sample preparation and analysis

**Fig. 1:**
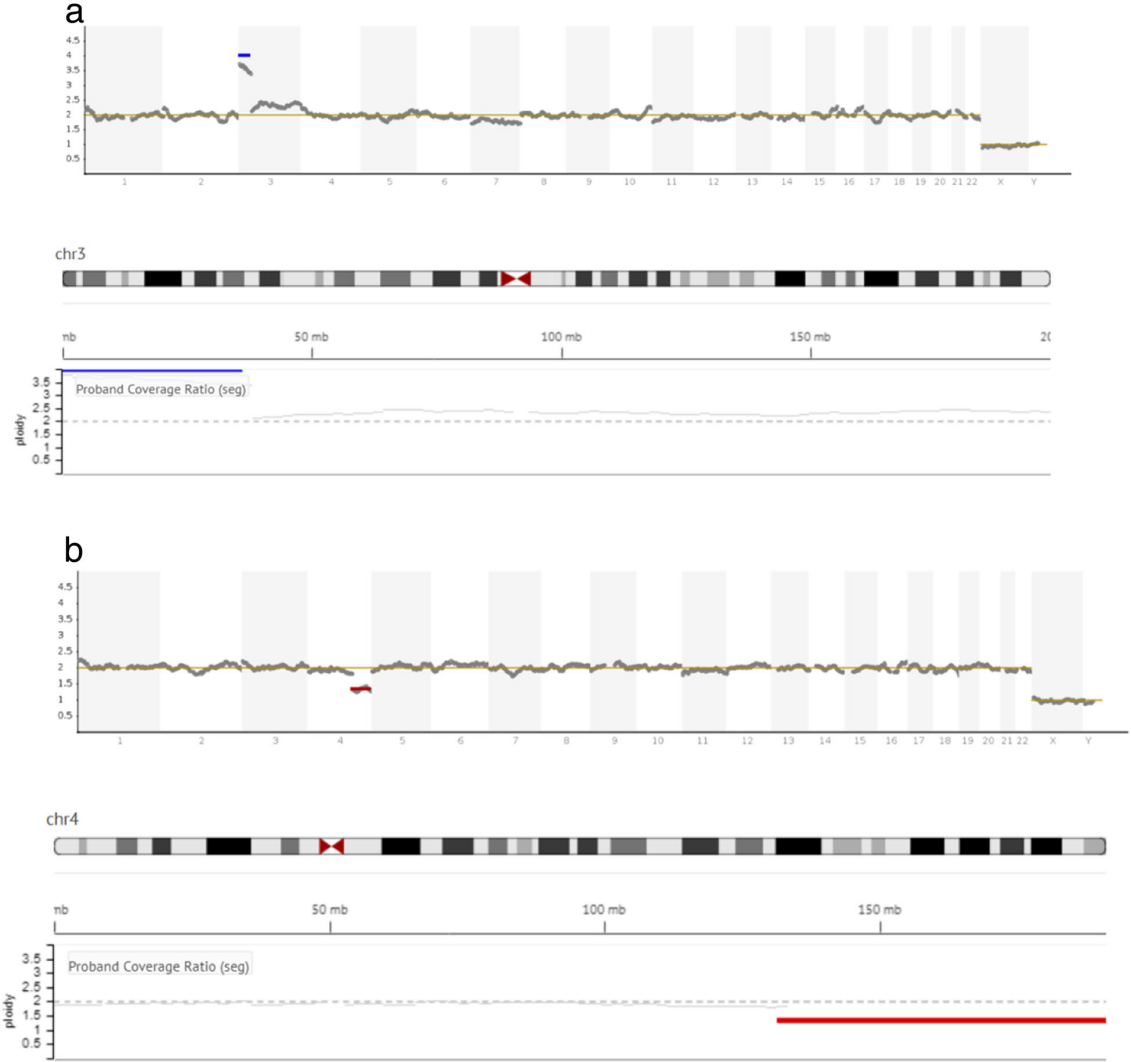
The graph represents the copy number variation (CNV) of the sample analyzed compared to the reference bioinformatics baseline. Data were generated from multiple normal samples using Ion Reporter software and were normalized using ReproSeq Low-Coverage Whole-Genome Baseline (5.0). **a**) shows a gain on the short arm of chromosome 3 (+3p). **b**) shows a loss on the large arm of chromosome 4 (−4q)

Sample preparation and genetic testing for aneuploidies were carried out according to the standard protocol at Igenomix (Valencia, Spain). In brief, biopsied TE cells were washed and placed in 5 μL PBS/1% (v/v) polyvinylpyrrolidone (Cell Signaling Technology, MA, USA), transferred to a 0.2 mL polymerase chain reaction (PCR) tube under sterile conditions, and stored at − 20 °C until analysis. DNA extraction and WGA were performed using the Ion ReproSeq TM PGS Kit (Thermo Fisher Scientific, USA). The kit/assay is used with the Ion Chef TM and Ion S5 System instruments (Thermo Fisher Scientific, Inc., MA, USA). Data were analyzed with Ion Reporter software 5.4, which aligns the readings with the human genome (hg19), which in turn uses the bioinformatic tool ReproSeq Low-pass whole-genome aneuploidy workflow v1.0, with low coverage (minimum 0.01x). An embryo was considered “abnormal” when an aneuploidy or partial aneuploidy was detected as a result of a deviation from the reference bioinformatics baseline (Fig. 1). Segmental aneuploidy was determined when a fragment of a chromosome > 5 Mb in size deviated from the standard thresholds for euploidy. This threshold is specifically defined by the manufacturer (see Ion Reporter™ 5.0 Software manual: https://assets.thermofisher.com/TFS-Assets/LSG/manuals/IonReporter_v50_Help.pdf). In short, corrected coverage is compared to a baseline of control samples with known correct ploidy. The normality threshold is stablished as two chromosomal copies for autosomes, and one for sexual chromosomes. The software computes a statistical model of the likelihood that a genomic region belongs to an alternate ploidy state.

### Variables and study design

Clinical data concerning patient age, medical indication for PGT-A and blastocyst biopsy (blastocyst stage, inner cell mass (ICM) and TE quality and biopsy day) were recorded. Morphological scoring for human blastocysts followed the system proposed by Cuevas et al. [27]. Analyzed blastocysts were classified as euploid or aneuploid. All aneuploid blastocysts were further classified as whole-chromosome or sub-chromosome (segmental) aneuploid embryos. Single or multiple segmental aneuploidies were recorded in association, or not, with whole-chromosome aneuploidy (see Results).

Clinical and embryological variables were compared in relation to genetic results. The database was reduced to 159 blastocysts, which were eventually classified as carriers of single segmental aneuploidies (SSA), affecting one single chromosome and no other aneuploidy was detected, pure-SSA. Data for pure-SSA blastocysts were re-analyzed from a qualitative and quantitative point of view. A novel aspect of this work is that only SSA blastocysts (without additional chromosomal abnormalities) were taken into account in this secondary analysis, since we felt that data for segmental or whole-chromosome aneuploidies could have obscured the topographic, qualitative and quantitative analyses. Qualitatively, SSA were described as gains or losses located on the small (p-) or large (q-) chromosome arm and on the affected chromosome. For quantitative terms, the DNA-sequence length (size, in Mb) of every SSA was described. Additionally, we described two indirect quantitative variables: (1) SSA length to total length of the carrier chromosome, including the centromere (namely, the SSA:chromosome ratio) and; (2) SSA length to total length of the arm of the affected chromosome (namely, the SSA:arm ratio). These chromosome and sub-chromosome lengths were obtained from the genome browser of Santa Cruz University of California (https://genome.ucsc.edu) and were used to calculate every SSA-ratio.

On the other hand, in order to analyze SSA distribution per chromosome throughout a karyotype, SSA were classified following the Denver System [28]. This standard system classifies chromosomes according to three parameters (summarized in McGowan-Jordan et al. [29]: 1) the length of each chromosome relative to the total length of a normal haploid set, expressed as a percentage of the total length of a normal haploid set (the sum of the 22 autosomes and the X chromosome lengths); 2) the arm ratio of the chromosomes, expressed as the length of the longer arm relative to the shorter one; and 3) the “centromeric index”, expressed as the ratio of the shorter arm length to the length of the whole chromosome. The latter two indices are related algebraically. Moreover, this standard chromosome classification confers importance to the chromosome sequence and heterochromatin distribution, rather than the size of each chromosome per se (Patau, 1960). Thus, seven chromosome groups (named A to G) were defined: group A includes large-sized metacentric or sub-metacentric autosomes (chromosome 1, 2 and 3); group B refers to large-sized sub-metacentric autosomes (chromosome 4 and 5); group C refers to medium-sized sub-metacentric autosomes (chromosomes 6–12 and X); group D refers to medium-sized acrocentric autosomes (chromosomes 13, 14 and 15); group E refers to small-sized metacentric or sub-metacentric autosomes (chromosomes 16, 17 and 18); group F refers to small-sized metacentric chromosomes (number 19 and 20); and group G refers to the smallest acrocentric chromosomes (21, 22 and Y).

### Statistical analysis

Categorical variables were expressed as number of cases (n) and percentage of occurrence (%). By means of the Chi-square test, applying Yates’s correction for continuity when appropriate, we compared the proportion of each SSA type and the chromosome involved according to the medical indication for PGT-A, blastocyst stage, ICM and TE quality, and biopsy day. Statistical significance was established at a *P*-value of 0.05.

Continuous variables (patient age, SSA size and the SSA:chromosome and SSA:arm ratios) were checked for normality using the Kolmogorov-Smirnov test. The data regarding age were adjusted to a normal distribution and analyzed using the analysis of variance test (ANOVA) according to clinical and embryological variables, as well as SSA type and chromosome affected. Data were presented as mean and standard deviation, with a 95% confidence interval (95% CI) when appropriate. Otherwise, data relative to SSA size were not adjusted to a normal distribution; thus, this continuous variable was transformed into a categorical one by re-grouping it into quartiles, thus providing us with four categories. In this sense, this transformed variable was compared to cited categorical variables by Chi-square tests, applying Yates’s correction for continuity when appropriate. Statistical significance was established at a *P*-value of 0.05.

All statistical analyses were performed using the Statistical Package for the Social Sciences 19.0 (SPSS, Chicago, IL, USA).

## Results

The results of 822 PGT-A cycles were analyzed. Table 1 shows data relating to number of cases, medical indication for PGT-A and patient age, which ranged from 22 to 46 years (average: 38.8 + 3.2 years; 95CI: 38.6–39.0). Table 2 shows the results of genetic testing for aneuploidies. Forty-six percent of blastocysts (1656 of 3565) were euploid, with incidence varying significantly according to PGT-A indication (Table 2). The remaining blastocysts were diagnosed as aneuploid (53.5%; 1909 of 3565). In 45.2% (1610 of 3565) of diagnosed blastocysts one (29.2%) or more (16.0%) whole-chromosomes were implicated in the aneuploidy.

**Table 1:** Number (and percentage, %) of cycles studied per clinical indication and maternal age

Clinical indication	Number of cycles (%)	Age (years)	
		Mean age ± standard deviation	Confidence interval: 95%
Recurrent miscarriage	74 (9.0)	36.7 ± 3.7^a^	35.8–37.5
Advanced maternal age	537 (65.3)	40.3 ± 1.8^b^	40.2–40.5
Prior chromosome pathology	19 (2.3)	34.8 ± 2.7^d^	34.6–36.5
Male factor	64 (7.8)	35.4 ± 3.1^c,d^	34.7–36.2
Implantation failure	120 (14.6)	35.5 ± 3.0^c,d^	35.0–36.0
Sperm aneuploidy	8 (1.0)	38.0 ± 2.3^a,b,c^	36.0–39.9
Total	822		

^a,b,c^Different superscripts within a column indicate significant differences (*P* < 0.05)

**Table 2:** Number and percentage (%) of euploid and aneuploid blastocysts. Blastocysts were classified according to clinical indication for preimplantational genetic test for aneuploidies (PGT-A) and by genetic categories: euploids, whole-chromosome aneuploids and segmental aneuploids (with or without whole-chromosome aneuploidy associated). Data from blastocysts presenting only one segmental aneuploidy (without any additional whole-chromosome gain/loss) were classified as single segmental aneuploid (SSA)

Clinical indication (n)	Number of euploid blastocysts (n, %)	Number of aneuploid blastocysts (n, %) with:				
		One or more whole-chromosome aneuploidies*	Segmental aneuploidy			
			Without whole-chromosome aneuploidies associated		With one or more whole-chromosome aneuploidies associated	
			One segmental aneuploidy (SSA)	> 1 segmental aneuploidy	One segmental Aneuploidy	> 1 segmental aneuploidy
Recurrent miscarriage (453)	245 (54.1) ^a^	169 (37.3) ^b^	21 (4.6)	4 (0.9)	13 (2.9)	1 (0.2)
Advanced maternal age (1778)	623 (35.0) ^b^	1011 (56.9) ^a^	67 (3.8)	3 (0.2)	67 (3.8)	7 (0.4)
Prior chromosome pathology (208)	126 (60.6) ^a,c^	66 (31.7) ^b,c^	12 (5.8)	0	4 (1.9)	0
Male factor (457)	293 (64.1) ^c^	127 (27.8) ^c^	22 (4.8)	3 (0.6)	11 (2.4)	1 (0.2)
Implantation failure (570)	314 (55.1) ^a^	207 (36.3) ^b^	27 (4.7)	3 (0.5)	17 (2.9)	2 (0.3)
Sperm aneuploidy (99)	55 (55.6) ^a,c^	30 (30.3) ^b,c^	10 (10.1)	0	3 (3.0)	1 (1.0)
Total (3565)	1656 (46.4)	1610 (45.2)	159 (4.5)	13 (0.4)	115 (3.2)	12 (0.3)

*Only whole-chromosome aneuploidy, no additional segmental aneuploidy

^a,b,c^ Different superscripts within a column indicate significantly statistical differences (*P* < 0.05)

In the case of segmental aneuploidy (Table 2), 8.4% of diagnosed blastocysts (299 of 3565) exhibited one or more segmental chromosome aneuploidies, some of which were associated with whole-chromosome aneuploidy, while others were not. Two-hundred and seventy-four of the 3565 blastocysts (7.7%) had a single segmental aneuploidy (SSA), associated (*n* = 115) or not (*n* = 159) with a whole-chromosome aneuploidy, whereas 0.7% of the remaining blastocysts (25 of 3565) showed segmental aneuploidies in two different chromosomes. Only one blastocyst was diagnosed as carrying three segmental aneuploidies located in three different chromosomes (multiple segmental aneuploidy). No more than three segmental aneuploidies per embryo, or one segment per chromosome and embryo, were observed.

Single segmental aneuploidies in the absence of whole-chromosome aneuploidies (pure-SSA) were detected in 159 blastocysts (4.5% analyzed blastocysts), independently of the medical indication for the assisted reproductive technology (ART) cycle (*P* > 0.05; Table 2).

Frequency of pure-SSA (*n* = 159) was not related to day of blastocyst biopsy (day 5 vs day 6; *P* = 0.70) or blastocyst stage (*P* = 0.58), while it was related to quality of the ICM and TE (*P* < 0.01). Thus, as shown in Table 3, a significantly higher percentage of pure-SSA was observed among blastocysts qualified as grade “C” (referring to TE and ICM) than among those with better TE and ICM quality.

**Table 3:** Number of pure single segmental aneuploid (SSA) or euploid blastocysts, according to the inner cell mass (ICM) and trophectoderm (TE) quality scores, ranged from high/good quality (“a/b”) to fair quality (named “c”)

Quality Score	Trophectoderm			Inner cell mass		
	“a”	“b”	“c”	“a”	“b”	“c”
Blastocyst with (n, %)						
SSA *n* = 159	1 (0.6)	70 (44.0)	88 (55.3)^a^	8 (5.0)	85 (53.5)	66 (41.5)^a^
Euploid *n* = 1661	58 (3.5)	1027 (61.8)	577 (34.7)^b^	166 (10.0)	1057 (63.3)	438 (26.4)^b^
	(3.2)	(60.2)	*P* < 0.01	(9.5)	(62.7)	*P* < 0.01
	*n* = 1821			*n* = 1821		

^a,b^Different superscripts within a column indicate significant differences in the percentage of blastocysts as SSA-carriers or euploid, according to a particular the ICM or TE quality score (*P* < 0.05)

From a qualitative point of view, we described the SSA population according to location of gains or losses on the p- or q-chromosome arms. In general, both gains (44.0%) and losses (56.0%) were equally represented in the pure-SSA population; however, they were more frequently located on the q- than on the p-chromosome arm (67.3% vs 32.7%, respectively). Moreover, SSA type, defined by combining both variables (gains/losses and arm location), was equally distributed in the blastocyst population (Table 4).

**Table 4:** Distribution of blastocysts with gains (+) or losses (−) on p- and q-chromosome arms, in function of day of trophectoderm biopsy. Percentages are in brackets

Day of biopsy	-p	-q	+p	+q	Total
Day 5	14 (8.8)^b^	22 (13.8)^b^	12 (7.5)^b^	35 (22.0)^a^	83 (52.2)
Day 6	18 (11.3)^b^	35 (22.0)^a^	8 (5.0)^b^	15 (9.4)^b^	76 (47.8)
Total	32 (20.1)	57 (35.8)	20 (12.6)	50 (31.4)	159

^a,b^Different superscripts within a column indicate significantly statistical differences (*P* < 0.05)

SSA type was not statistically affected by age (*P* = 0.51), clinical indication (*P* = 0.15), blastocyst stage (*P* = 0.54) or ICM and TE quality (*P* = 0.2 and *P* = 0.28, respectively), but it was significantly affected by day of biopsy (*P* = 0.007; Table 4). Thus, blastocysts biopsied on day 5 showed significantly higher percentages of gains on the q-chromosome arms (22.0%), whereas those biopsied on day 6 showed significantly higher percentages of SSA losses on the q-chromosome arm (22.0%). SSA affecting p-chromosome arms were equally distributed amongst blastocysts biopsied on day 5 or 6 of development (ranging from 5.0 to 11.3%; Table 4).

**Fig. 2:**
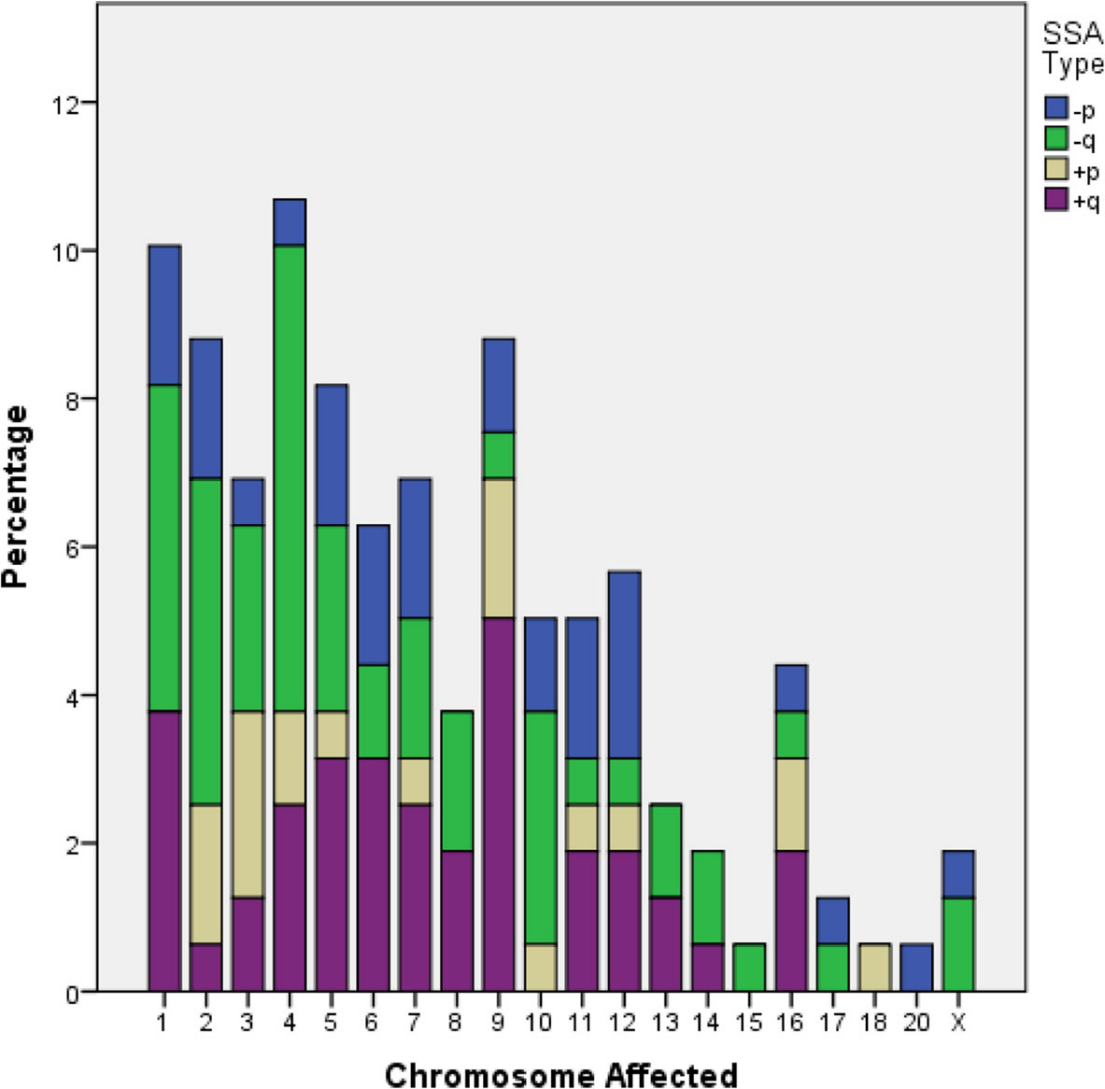
Percentage of blastocysts diagnosed as single segmental aneuploid (SSA) and types (losses on the small or large chromosome-arm: -p, −q, respectively; or gains on the small or large chromosome-arm: +p, +q, respectively) according to chromosome carrier

Qualitative description of SSA was also defined by the chromosome involved. The Kolmogorov-Smirnov test revealed that the frequency of the chromosomes with a SSA did not follow a normal distribution (Fig. 2; *P* < 0.001). In fact, our SSA population displayed an asymmetrical distribution of chromosome frequency: SSAs were located on chromosomes 1 to 9 in nearly two thirds of blastocysts, whereas 29.6% of SSA were located on the remaining autosomes and sexual chromosomes. No SSA was observed on the Y chromosome or on autosomes 19, 21 or 22 (Fig. 2).

Additionally, SSA affecting a particular chromosome was not statistically related with age (*P* = 0.92), medical indication (*P* = 0.24), day of biopsy (*P* = 0.25), blastocyst stage (*P* = 0.96) or ICM quality (which was constantly rated “b”). On the other hand, a significant relation was observed between TE quality and the affected chromosome (*P* = 0.04). No statistical analysis was performed to explore the relation of SSA size to the chromosome carrier due to the relatively low number of cases studied.

Interestingly, although current qualitative descriptions include both topographic location of gains/losses on the chromosome arm and the chromosome involved, our data showed that these two qualitative variables were not related to each other (*P* = 0.09; Fig. 2; Table 5).

**Table 5:** Number and percentage (within brackets) of every qualitative type of single segmental aneuploidy (SSA), according to the chromosome affected

Chromosome carrier of a SSA	SSA type (n, %)				TOTAL
	-p	-q	+p	+q	
Chromosome 1	3 (1.9)	7 (4.4)	0 (0.0)	6 (3.9)	16 (10.1)
Chromosome 2	3 (1.9)	7 (4.4)	3 (1.9)	1 (0.6)	14 (8.8)
Chromosome 3	1 (0.6)	4 (2.5)	4 (2.5)	2 (1.3)	11 (6.9)
Chromosome 4	1 (0.6)	10 (6.3)	2 (1.3)	4 (2.5)	17 (10.7)
Chromosome 5	3 (1.9)	4 (2.5)	1 (0.6)	5 (3.1)	13 (8.2)
Chromosome 6	3 (1.9)	2 (1.3)	0 (0.0)	5 (3.1)	10 (6.3)
Chromosome 7	3 (1.9)	3 (1.9)	1 (0.6)	4 (2.5)	11 (6.9)
Chromosome 8	0 (0.0)	3 (1.9)	0 (0.0)	3 (1.9)	6 (3.8)
Chromosome 9	2 (1.3)	1 (0.6)	3 (1.9)	8 (5.0)	14 (8.8)
Chromosome 10	2 (1.3)	5 (3.1)	1 (0.6)	0 (0.0)	8 (5.0)
Chromosome 11	3 (1.9)	1 (0.6)	1 (0.6)	3 (1.9)	8 (5.0)
Chromosome 12	4 (2.5)	1 (0.6)	1 (0.6)	3 (1.9)	9 (5.7)
Chromosome 13	0 (0.0)	2 (1.3)	0 (0.0)	2 (1.3)	4 (2.5)
Chromosome 14	0 (0.0)	2 (1.3)	0 (0.0)	1 (0.6)	3 (1.9)
Chromosome 15	0 (0.0)	1 (0.6)	0 (0.0)	0 (0.0)	1 (0.6)
Chromosome 16	1 (0.6)	1 (0.6)	2 (1.3)	3 (1.9)	7 (4.4)
Chromosome 17	1 (0.6)	1 (0.6)	0 (0.0)	0 (0.0)	2 (1.3)
Chromosome 18	0 (0.0)	0 (0.0)	1 (0.6)	0 (0.0)	1 (0.6)
Chromosome 19	0 (0.0)	0 (0.0)	0 (0.0)	0 (0.0)	0 (0.0)
Chromosome 20	1 (0.6)	0 (0.0)	0 (0.0)	0 (0.0)	1 (0.6)
Chromosome 21	0 (0.0)	0 (0.0)	0 (0.0)	0 (0.0)	0 (0.0)
Chromosome 22	0 (0.0)	0 (0.0)	0 (0.0)	0 (0.0)	0 (0.0)
Chromosome X	1 (0.6)	2 (1.3)	0 (0.0)	0 (0.0)	3 (1.9)
Chromosome Y	0 (0.0)	0 (0.0)	0 (0.0)	0 (0.0)	0 (0.0)
TOTAL	32 (20.1)	57 (35.8)	20 (12.6)	50 (31.4)	159

No significant relation was observed between both qualitative variables (chromosome affected and type of SSA); *P* = 0.109

Description of SSA from a quantitative point of view requires the study of DNA-sequence length (Additional file 1). The Kolmogorov-Smirnov test revealed that SSA size did not follow a normal frequency distribution (*P* < 0.001). Thus, this continuous variable (SSA size) was converted to a categorical one by re-grouping the sizes into quartiles in order to perform statistical comparisons with continuous variables such as patient age.

**Fig. 3:**
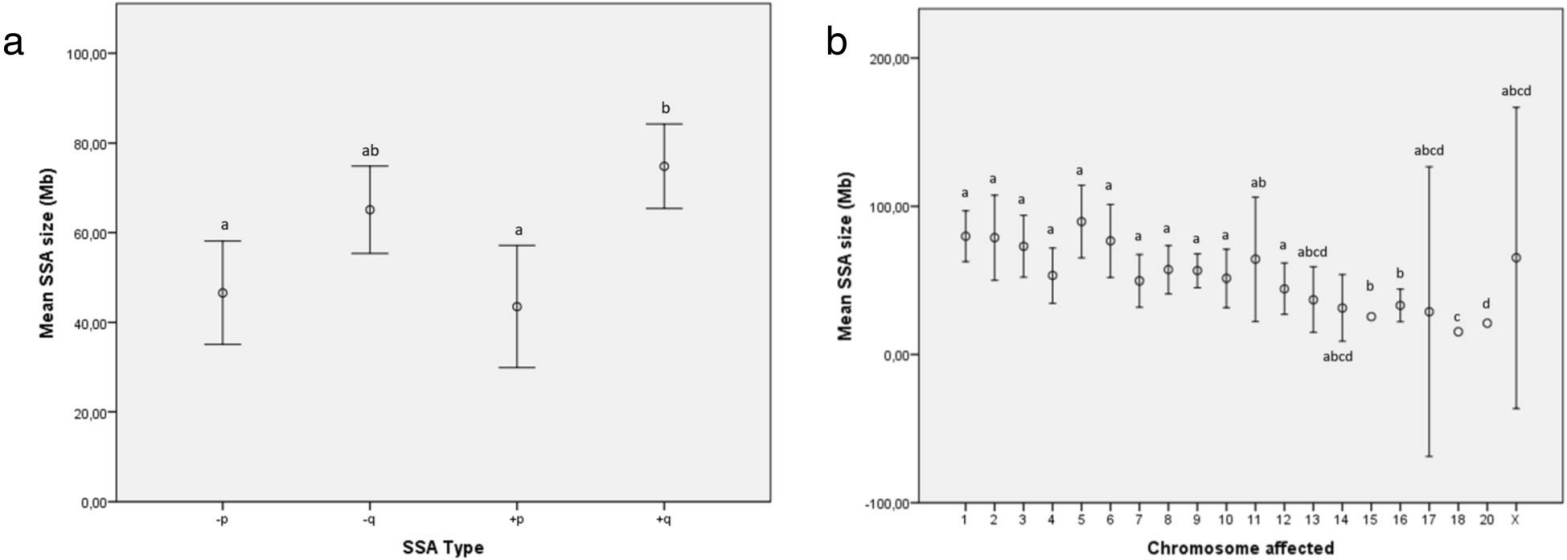
Average SSA size (Mb; open circle) according to SSA type (Fig. 3**a**) and chromosome carrier (Fig. 3**b**). Error bars represent 95% confidence interval in Mb. Footnote: Different superscripts represent statistically significant differences (*P* < 0.05) between SSA types or affected chromosomes

SSA size was not statistically related to age (*P* = 0.99), medical indication (*P* = 0.48), day of biopsy (*P* = 0.18), blastocyst stage (*P* = 0.40), or TE (*P* = 0.09) or ICM quality (constantly rated “b”). However, significant differences were observed according to SSA type (*P* = 0.003) and the chromosome involved (*P* = 0.007). Thus, gains and losses located on the p-arm had comparable average sizes (45.4 ± 30.6 Mb; 95CI: 36.9–53.9 Mb; *P* = 0.99) and were significantly shorter than gains on the q arm (average: 74.8 ± 33.2 Mb; 95CI: 65.4–84.2 Mb; *P* < 0.03), whereas losses on the q-arm were of an intermediate size (average: 65.1 ± 36.9 Mb; 95CI: 55.3–74.9 Mb; Fig. 3a).

Otherwise, SSA size was related to the chromosome involved (*P* = 0.003; Fig. 3b). However, since the analysis of SSA size in relation to chromosome rendered a relatively low number of cases, no further analysis was performed.

**Fig. 4:**
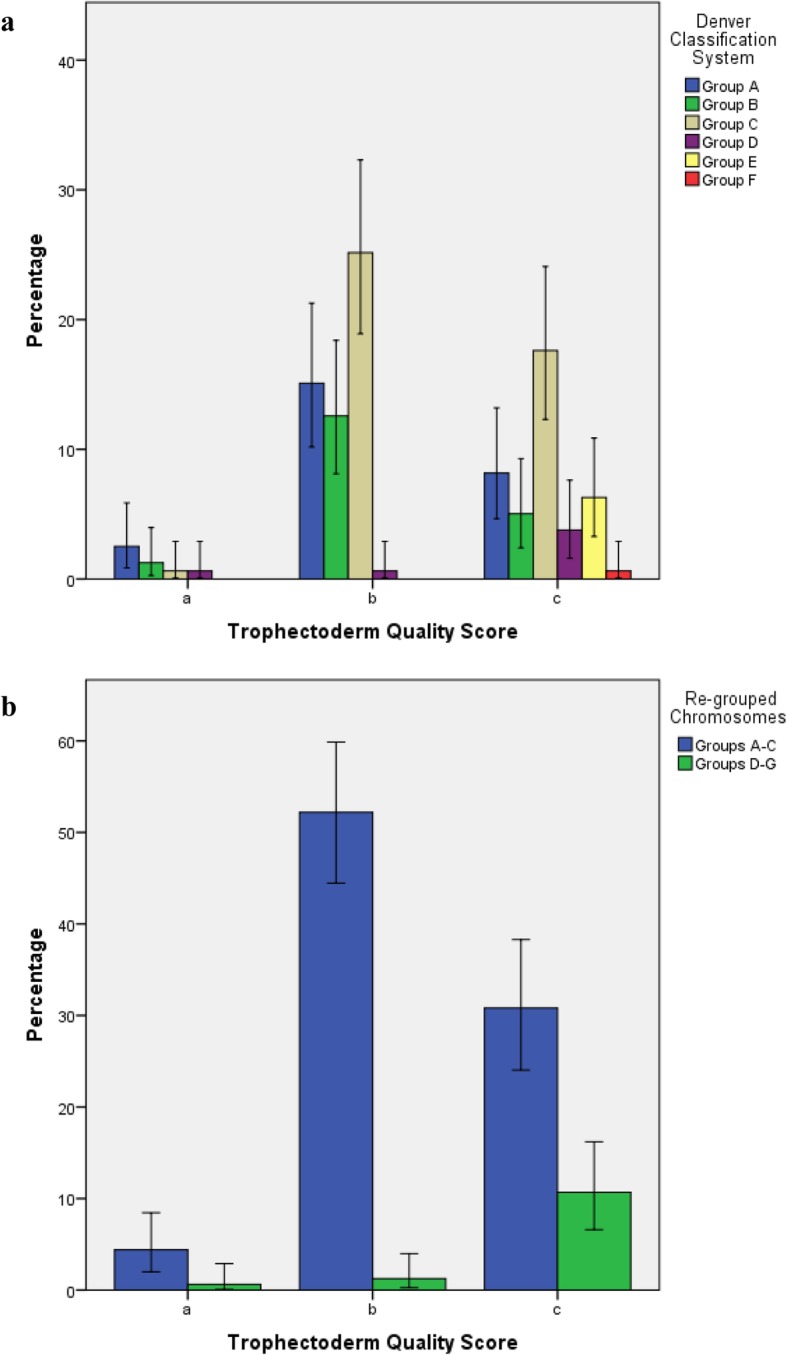
Percentage (mean, error bars: 95% confidence interval) of pure SSA located in each Denver chromosome classification group, according to excellent, good or fair trophectoderm quality scores (a, b and c, respectively; Fig. 4**a**). Lower figure shows percentage (mean error bars: 95% confidence interval) of SSA located in chromosomes pertaining to groups A-C or D-F, according to trophectoderm quality scores (Fig. 4**b**)

The above mentioned relationship between TE quality and affected chromosome was also observed after grouping chromosomes according to the Denver classification. Thus, in fair TE quality blastocysts (rated “c”), significantly more pure-SSA were observed on acrocentric or small–sized chromosomes (Groups D-F) than in blastocysts with excellent or good TE quality (rated “a” or “b”), in which such chromosomes were rarely affected (*P* = 0.00006; Fig. 4). Pure SSA were more frequently located on large- or medium-sized sub-metacentric chromosomes (Group A-C), regardless trophectoderm quality (average: 88.0%; *P* = 0.25; Fig. 4).

**Fig. 5:**
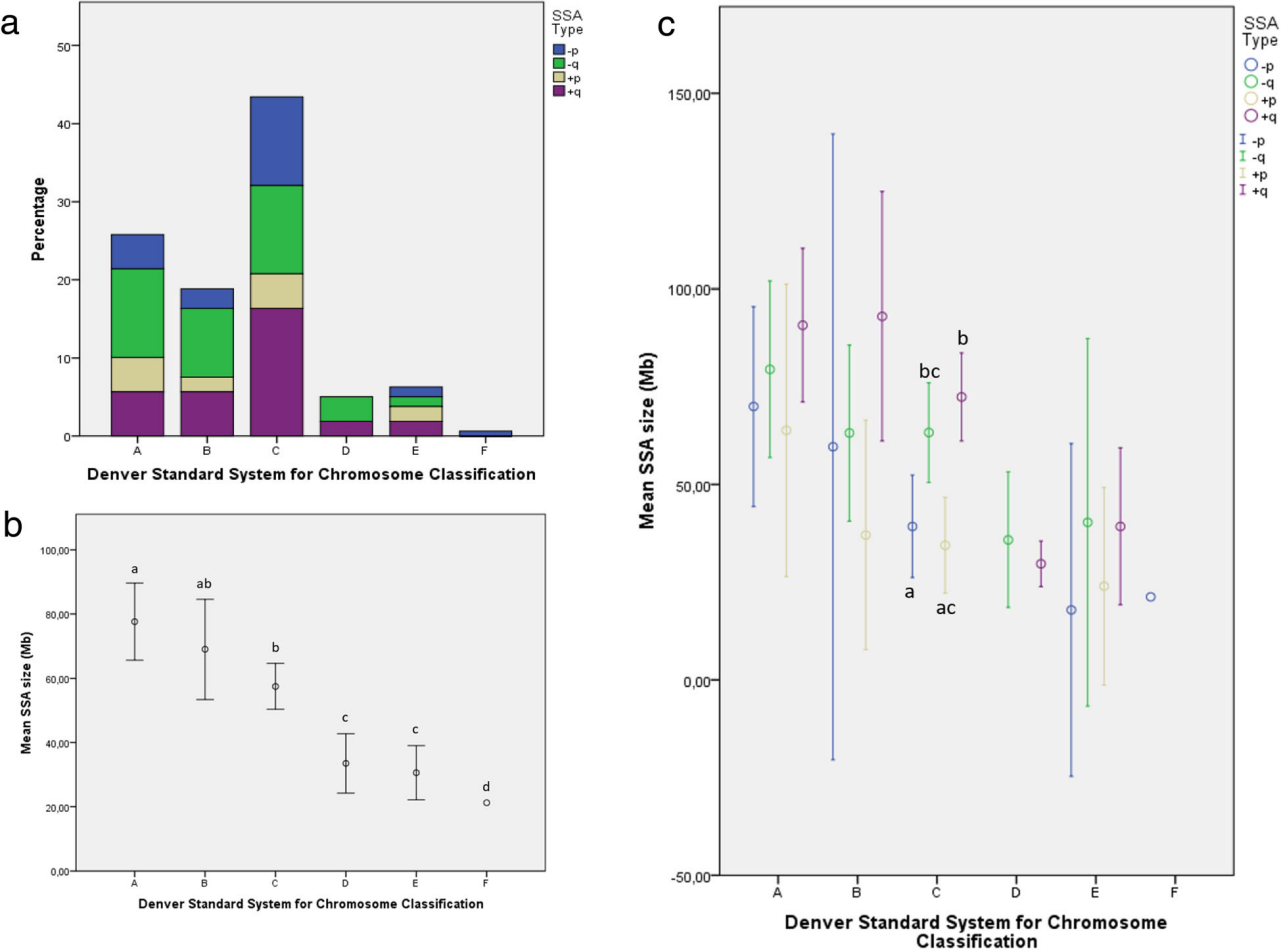
Frequency of single segmental aneuploid (SSA) blastocysts, with detailed types of SSA (Fig. 5**a**) and average mean SSA sizes (open circles; Mb, Fig. 5**b**) according to the Denver classification system. Averaged mean size (open circles) of each SSA type classified by the Denver Standard System (Fig. 5**c**). Error bars represent 95% confidence interval in Mb. Footnote: Different superscripts represent statistically significant differences (*P* < 0.05) in SSA size between chromosome categories or SSA types within C-chromosome category

Nevertheless, we re-grouped the SSA population according to the Denver Standard chromosome classification system [26]. As shown in Fig. 5a, pure SSAs were most frequently represented in group C (43.4%), followed by those in groups A (25.8%) and B (18.9%). The remaining blastocysts exhibited a SSA in chromosomes in groups D (5.0%) and E (6.3%), and only one blastocyst had SSA in chromosome 20 (group F: 0.6%). No SSA was detected in the small-sized metacentric chromosome 19 or acrocentric chromosomes 21, 22 and Y (Fig. 2 and Fig. 5a). Following the Denver Standard System, different SSA sizes were observed amongst the chromosome categories analyzed (*P* = 0.0001; Fig. 5b). Thus, although comparable sizes were observed amongst groups D and E (31.9 ± 11.3 Mb; 95CI: 26.3–37.5 Mb), they were significantly shorter than those in groups A-C and larger than the single SSA observed in chromosome 20 (group F: 21.2 Mb). Moreover, SSA sizes in group A were significantly larger (average: 77.6 ± 38.1 Mb; 95CI: 65.6–89.6 Mb) than those in medium- or small-sized chromosomes, regardless of the centromere emplacement (groups C-G). The SSAs identified in large-sized sub-metacentric autosomes (group B: 69.0 ± 41.7 Mb; 95CI: 53.4–84.6 Mb) were of an intermediate size with respect to groups A (large-size) and C (medium-sized sub-metacentric chromosomes; average: 57.5 ± 29.6 Mb; 95CI: 50.4–64.6 Mb), but were significantly larger than those quantified in small-sized chromosomes (groups E-F) and medium-sized acrocentric chromosomes (group D).

Additionally, we assessed the size of all four SSA types within each chromosome group (Fig. 5c). The results showed comparable SSA sizes regardless of SSA type in all groups except for group C. In this way, sequences corresponding to gains or losses on the q-arm were significantly larger (average: 68.6 ± 27.1 Mb; 95CI: 60.4–76.8 Mb) than losses on the p-arm (average: 39.2 ± 26.8 Mb; 95CI: 26.2–52.3 Mb). Gains on the p-arm were of an intermediate size with respect to SSA losses, whatever the chromosome arm affected (average: 34.4 ± 13.3 Mb; 95CI: 22.1–46.7 Mb).

**Fig. 6:**
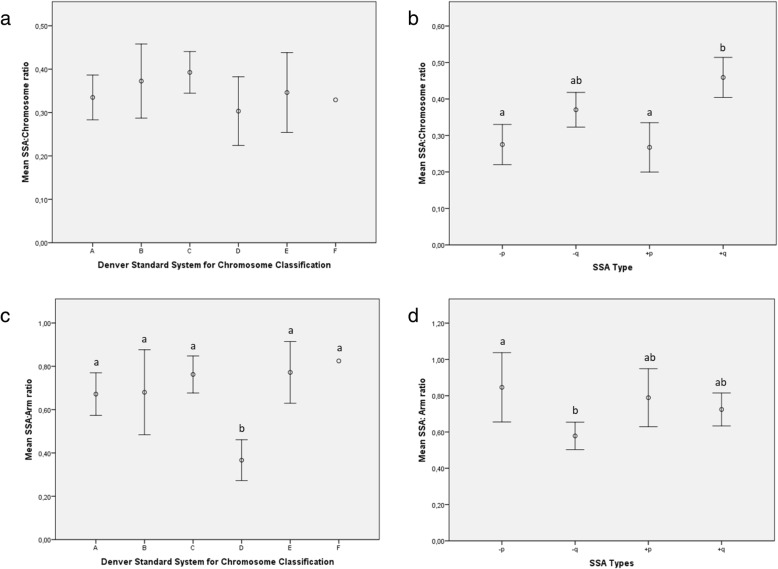
Average SSA:chromosome ratio (open circles; ranged 0–1) according to the Denver classification system (Fig. 6**a**) and SSA type (Fig. 6**b**). Average SSA:arm ratio (open circles; ranged 0–1) according to the Denver classification system (Fig. 6**c** and SSA type (Fig. 6**d**). Error bars represent 95% confidence interval in Mb. Footnote: Different superscripts represent statistically significant differences (*P* < 0.05) in the SSA:chromosome or SSA:arm ratio between chromosome categories or SSA type

Finally, we calculated the ratio of SSA size according to the length of the entire chromosome, including the centromere (SSA:chromosome ratio). The results showed that the SSA:chromosome ratio was pretty much constant for all the chromosome groups classified according to the Denver Standard System (*P* = 0.62), with an estimated average of 0.37 ± 0.19 (95CI: 0.37–0.40; Fig. 6a). However, the SSA:chromosome ratio was affected by SSA type (*P* < 0.001; Fig. 6b); SSAs on the p-arms had a significantly lower ratio (average: 0.27 ± 0.15; 95CI: 0.23–0.31) than gains on the q-arms (average: 0.46 ± 0.19; 95CI: 0.40–0.51). An intermediate ratio (0.37 ± 0.18; 95CI: 0.32–0.42) was calculated for losses on the q-chromosome arm.

The ratio of SSA size to arm length (SSA:arm ratio) was comparable in almost all the chromosome groups when the Denver Standard classification was employed; an average ratio of 0.72 ± 0.37 was obtained (95CI: 0.66–0.78; *P* = 0.71; Fig. 6c), with the exception of group D, in which it was significantly lower (average: 0.37 ± 0.11; 95CI: 0.27–0.46). SSA:arm ratios were also affected by SSA type (*P* = 0.005; Fig. 6d). Thus, losses on the q-arm showed significantly lower SSA:arm ratios (average: 0.27 ± 0.15; 95CI: 0.22–0.33) than those located on the p-arm (average: 0.37 ± 0.18; 95CI: 0.32–0.42), while gains displayed intermediate SSA:arm ratios, whatever the chromosome arm affected (averaged: 0.74 ± 0.32; 95CIs: 0.66–0.82).

## Discussion

PGT-A has revealed different grades of chromosomal instability in preimplantation embryos. The concept of “chromosomal instability” (CI) was described by Geigl et al. [30] as “*the rate (cell-to-cell variability) of gain or loss of whole chromosomes or fractions of chromosomes”,* a definition that *“encompasses the rate of both whole-chromosome and segmental chromosomal aneuploidies”* [31]. In line with this idea, the detection of intrachromosome segments is highly important, even if the frequency, type, DNA-sequence size and chromosomal distribution of these changes are not well understood. According to the literature their prevalence varies between 4 and 58% (reviewed by Treff and Franasiak, [3]).

In our dataset, segmental aneuploidy was detected in 8.6% of trophectoderms, in accordance with previous reports [18, 23]. We did not detect any relation between the rate of segmental aneuploidies and medical indication for ART. In addition, no relationship was found between incidence of segmental aneuploidy and the day of blastocyst biopsy (day 5 vs day 6) or blastocyst stage. However, the rate of segmental aneuploidy was significantly higher among blastocysts with poor ICM and TE quality, which is one of the novel findings of the present work.

No correlation was found between SSA type and maternal age, medical indication, blastocyst stage, or ICM and TE quality, while a correlation was detected with the day of biopsy, as those performed later (day 6) showed an increased percentage of losses on the q-chromosome arm.

In general, more segments were observed on the q-arm than on the p-arm. Thus, the presence of SSA and its type could be related to either the minimal length of the arm with the SSA - which is supported by the absence of p-arm SSA in groups D and F - or chromosome length. Alternatively, we may be faced with a question of varying origins of the segments, but that is an aspect which goes beyond the scope of this study. On the other hand, the frequency of gains and losses was similar, which suggests a stochastic biological event that can occur with equal probability in either of the arms.

Concerning chromosome type, the highest rate of SSA was detected in chromosomes 1 to 9, which are the largest of their kind (in absolute values, in Mb). This rate was not related with any of the previously cited medical or embryonic parameters. To explore the chromosomal factor further, we studied the incidence of SSA per chromosome and found that SSAs were most clearly associated with chromosome group C, followed by groups A and B. The absence of SSA in chromosomes 19 and 22 is in line with Babariya et al. [23], who reported a drastic decrease in the incidence of segmental aneuploidy in both chromosomes between the cleavage and blastocyst stages. A dramatic fall in the percentage of segmental aneuploidies in chromosome 19 was observed in TE samples. Chromosome 19 is the most gene-dense chromosome in the human genome [32], which has led to the hypothesis that it is affected by a selective pressure [32, 33]. The fact that none of our blastocysts was diagnosed with a SSA on chromosome 19 might be related with extremely poor embryo development prognosis; in this sense, only non-fragmented chromosome 19 embryos will be capable of reaching the blastocyst stage. This hypothesis is in line with reports by other groups who have related a high rate of arrested embryo development with aneuploidies on chromosome 19 [34].

Similarly, segmented acrocentric chromosomes (21, 22 and Y) might be subject to selection pressure [33]; alternatively, the smaller size of such chromosomes and the limits of detection could explain why they were not affected by SSA.

On the other hand, on a quantitative scale, the length of the DNA sequence implied in SSA size was not related with any of the parameters studied (maternal age, clinical indication, day of biopsy, blastocyst stage, or ICM and TE quality). However, in absolute values, segmental size depended on the chromosome involved, as well as on the arm. SSA size decreased progressively from large chromosomes to medium- or small-sized chromosomes. However, the ratio of SSA size to entire chromosome size (SSA:chromosome ratio) seemed to be constant and never exceeded 50% of the chromosome, except for medium-sized acrocentric chromosomes (group D), which were significantly shorter, probably due to the absence of SSA on the p-arm.

In absolute values, segments located on the q-chromosome arm were larger than those on the p-arm and were also longer in relation to full chromosome length (SSA:chromosome ratio); however, the opposite can be said for segmental size with respect to chromosome arm (SSA:arm ratio). In this way, segments located on the q-arm were shorter than those on the p-arms, which suggests a compensatory behavior of segmental aneuploidy in function of its position on the chromosome.

The literature addressing the genesis of segmental aneuploidy is scarce, but the available evidence suggests that the mechanisms implicated in segmental aneuploidy differ from those put forward to explain whole-chromosome numerical abnormalities or mosaicism. In general, segmental aneuploidy appears to be related to disturbances during mitosis and is independent of maternal age, as we and other authors have observed [35]. Chromosomal breakage, centric fission with missagregation of the telocentric fragment, and formation of ring chromosomes or i(p) or i(q) isochromosomes have been proposed as possible origins of segmental aneuploidy [36]. It has also been suggested that terminal segmental imbalances are a result of DNA double-stranded-breaks and non-disjunction of the acentric fragment, resulting in duplication of the remaining chromosomal segment [31]. Truncated chromosomes could be rescued by fusion of replicated sister chromatids, producing dicentric isochromosomes which can be separated by monopolar or bipolar segregation, producing, in turn, pure terminal imbalances and deletions with inverted duplications by a breakage-fusion-bridge-based mechanism [31].

The grade of chromosomal instability in preimplantation human embryos is usually related to that implicated in neoplastic events. Some biological features, such as rapid kinetics (especially in TE cells), short cell cycles, relaxation of mitotic checkpoints and deregulated apoptotic mechanisms, appear in both neoplastic and embryonic cells. These traits challenge a correct chromosome dosage. However, said features are also crucial for preimplantation embryos to achieve optimal size and morphology [37, 38]. In fact, a relatively new phenomenon known as chromothripsis has been highlighted as a potential cause of segmental chromosome imbalances in preimplantation embryos [39, 40]. In short, chromothripsis is a proposed mechanism consisting of a single-step event in which double-strand breaks occur in one or several chromosomes. These breaks generate chromosomal fragments that can be re-assembled during subsequent interphases, generating derivative chromosome(s) and additional small acentric or dicentric fragments [41, 42]. In a scenario of rapid cellular kinetics, characteristic of preimplantation embryos (and tumor cell lines), such chromosome fragments may be reassembled via inaccurate DNA repair mechanisms (homologous recombination and non-homologous end joining). Finally, during diakinesis, such acentric chromosome fragments, which lack proper kinetochores, would be incapable of segregating correctly and would eventually be transmitted (randomly) to daughter cells in both cleave-stage embryos and blastocysts [43]. Thus, individual chromosomes, or fragments, may be captured within small nucleus-like structures or micronuclei [44, 45], which can be identified in human preimplantation embryos by new time-lapse culture platforms [45, 46]. In accordance with Babariya et al. [23], the higher rate of chromosomal instability observed in cleave-stage embryos vs. blastocysts is compatible with a selection process against non-euploid embryos. However, the erratic distribution of chromosome segments does not completely arrest embryo development at the blastocyst stage. In this sense, it appears that there is a certain “mitotic permissiveness” towards inappropriate chromosomal segregation in early preimplantation stages, whose pathological significance might depend on the chromosomal content of the lost/gained fragment.

Another point for discussion is the assumption that segmental aneuploidy is a misdiagnosis related to the cell-cycle stage of biopsied cells. It is well established that DNA duplication before cell division is critical to ensure genome stability. DNA replication begins at locations of the genome called replication origins (ROs), which tend to be grouped in clusters along the chromosome and whose activation occurs stochastically in the early S-phase. The competence, efficiency and timing of ROs are key parameters that regulate replication behavior [47, 48]. Newly synthesized DNA in a single blastomere has been suggested as a source of error in PGT-A [20, 49]. Following on from this idea, a single blastomere from human cleavage-stage embryos could be “over-diagnosed”, since DNA replication domains can generate CNV changes that resemble segmental aneuploidy. This situation is not easily recreated in TE biopsied samples in which the cells are unlikely to all be in the same phase of the cell cycle, much less in the same phase of DNA replication. Instead TE samples are likely to be formed by mixed populations of cells in which G0/G1-cells predominate [18]. Nevertheless, it has been demonstrated in a mixed-cell model that the presence of few S-phase cells does not interfere with chromosomal copy number detection [20].

Our knowledge of the dynamics of TE cell growth is patchy, but rapidly dividing cells such as TE (and tumor cells) are usually in the S-phase and can be at varying stages of DNA replication. This hypothetical situation would translate as segmental aneuploidy mosaicism [3] and would confirm the crucial need for effective embryo biopsy techniques and trophectoderm quality. Indeed, the role of biopsy method should not be ignored, since an excessive use of laser pulses very close to the TE hernia can harm cells (thermolysis), reducing the number of cells in the sample and undermining the genetic quality of the biopsied sample. Thus, blastocyst manipulation can transform a sample of several trophectoderm cells into a “single-cell” sample open to the bias typically associated with single-cell diagnosis, including the mistaking of artifacts for segmental aneuploidies [50].

In the present work, we have related SSA to poor embryo prognosis (TE and ICM quality) and slow division (late biopsy). Poor embryo prognosis has recently been associated with oocyte- and cleavage-stage embryos with high rates of complex segmental aneuploidies, which have been attributed to hypothetical iatrogenic factors in IVF laboratories [23]. Indeed, blastocyst euploidy rates [1] and segmental abnormality variations [51] have been clearly linked to particular IVF centers. In line with such evidence, and in endorsement of the work of other authors [2], we suspect that the occurrence and type of segmental aneuploidy are internal quality indicators of IVF cycles, a hypothesis that requires testing in comparative inter-laboratory studies.

This observational study has some limitations associated with its retrospective nature. In addition, the causality of the correlations detected between the compared variables has not been investigated. However, in our view, such preliminary observational studies are relevant and necessary when a new phenomenon arises. We are confident that the analysis and discussion of SSA herein, together with previous reports by other authors [2, 3, 23], can help to design future prospective studies to determine the clinical significance of said phenomenon.

While we have used a single NGS-based platform for chromosomal analysis, a comparative study using different platforms could confirm our discoveries and offer answers to the questions raised by our data. Concerning technical limitations, the detection of mosaicism of segmental aneuploidy is pending validation with current technologies. According to a recent work by Zore and co-workers [52], studies in segmental mosaics are both limited and conflicting, and the limits of detection should be accurately stablished. For this reason, segmental aneuploidy mosaicism has not been considered in this paper. Finally, correlation studies between ICM and TE genetic constitution could help to throw further light on the biological effect of segmental aneuploidy.

## Conclusion

SSA in human blastocysts has been poorly studied until the recent implementation of NGS, and the biological basis for the underlying events is still not fully understood. Deregulated machinery of cell division, relaxation of mitotic control points, biopsy method and iatrogenic factors may be implicated in segmental aneuploidy. The clinical implication of these partial chromosomal gains/losses is unknown, as the epidemiological data on newborns are scarce and fragmentary. The prevalence of these sub-chromosomal abnormalities in the general population is infra-registered, and most cytogenetic analyses of fetuses or newborns are performed using low resolution techniques (karyotype or non-invasive NGS-based protocols). New data based on inter-laboratory ring-tests and prospective studies are vital to establish criteria for deciding whether or not affected embryos should be transferred, especially in extreme situations in which only one segmental-aneuploid embryo is available.

## Additional file

Additional file 1: This file shows the Embryo ID and corresponding breaking points of a gained (+) or lost (−) segment per chromosome. (DOCX 18 kb)

## Data Availability

The primary data for this study is available from the authors on direct request.

## Abbreviations

95% CI: 95% confidence interval
aCGH: Microarray-based Comparative Genomic Hybridization
ANOVA: Analysis of Variance
ART: Assisted reproductive technology
BACs: Bacterial Artificial Chromosomes
BOBs: BACs-on-beads
CGH: Comparative Genomic Hybridization
CI: Chromosomal instability
CN: Copy Number
CNV: Copy number Variation
DNA: Deoxyribonucleic acid
FISH: Fluorescence in situ Hybridization
hg: Human genome
ICM: Inner Cell Mass
ICSI: Intracytoplasmic sperm injection
IVF: In Vitro Fertilization
IVIRMA: Instituto Valenciano de Infertilidad Reproductive Medicine Associates
Mb: Megabase
NGS: Next Generation Sequencing
PBS: Phosphate-buffered saline
p-chromosome arm: Small chromosome arm
PCR: Polymerase Chain Reaction
PGT-A: Preimplantation Genetic Test for Aneuploidy
q-chromosome arm: Large chromosome arm
qRT-PCR: Quantitative real-time polymerase chain reaction
ROs: Replications Origins
SNP: Single Nucleotide Polymorphism
SPSS: Statistical Package for the Social Sciences
SSA: Single segmental aneuploidy
TE: Trophectoderm
vs: Versus
WGA: Whole Genome Amplification
