# Supplementary material for: Segmental aneuploidy in human blastocysts: a qualitative and quantitative overview
Source: Reprod Biol Endocrinol. 2019 Sep 16;17:76. doi: 10.1186/s12958-019-0515-6 (PMC6745804; doi:10.1186/s12958-019-0515-6)
Supplement: Supplementary file 1 — This file shows the Embryo ID and corresponding breaking points of a gained (+) or lost (−) segment per chromosome. (DOCX 18 kb) [file 12958_2019_515_MOESM1_ESM.docx]

Supplementary data

This file shows the Embryo ID and corresponding breaking points of a gained (+) or lost (-) segment per chromosome. Asterisked embryos (n=159) refers to those blastocysts diagnosed as a carriers of a single segmental aneuploidy.

| **Embryo ID** | **SEGMENT** |  |
| --- | --- | --- |
| **1** | **+3q24q29** |  |
| **2** | **+3q24q29** |  |
| **3** | **-5q33.3q35.3** |  |
| **4** | **-1p36.33p13.2** | * |
| **5** | **-7q21.12q36.3** |  |
| **6** | **-16p13.3p11.2** |  |
| **7** | **-3q13.32q29** | * |
| **8** | **-1p36.33p34.2** | * |
| **9** | **-7q12-q44** |  |
| **10** | **+1p36.33p31.1** |  |
| **11** | **+1q23.2q44** |  |
| **12** | **+1q21.2q44** | * |
| **13** | **+1q21.1q44** | * |
| **14** | **-2p25.3q22.1** |  |
| **15** | **-2q11.1q37.3** | * |
| **16** | **+2p23p25** | * |
| **17** | **+2q23.1q37.3** |  |
| **18** | **-3p26.3p24.1** | * |
| **19** | **-3q11.1q29** | * |
| **20** | **-3q11.1q26.33** |  |
| **21** | **+3p26.3p21.31** | * |
| **22** | **+3p26.3p24.2** |  |
| **23** | **+3q11.1q29** | * |
| **24** | **-4q33q35.2** | * |
| **25** | **-4q31.1q35.2** | * |
| **26** | **-4q28.1q35.2** |  |
| **27** | **-4q28.1q35.2** |  |
| **28** | **-5q11.1q23.1** |  |
| **29** | **-5p15.33p13.2** |  |
| **30** | **-5q14.3q35.3** |  |
| **31** | **-5q13.3q35.3** | * |
| **32** | **+5q11.1q35.3** | * |
| **33** | **+5p15.33p13.2** | * |
| **34** | **+5q15q35.3** |  |
| **35** | **+5q15q35.3** |  |
| **36** | **+5q14.3q35.2** |  |
| **37** | **+6q16.1q27** |  |
| **38** | **+7p21.3p22** | * |
| **39** | **+7q31.1q36.3** | * |
| **40** | **-9q21.11q34.3** |  |
| **41** | **+9p24.3p13.1** |  |
| **42** | **+9p24.3p13.1** |  |
| **43** | **-12q21.2q34.3** | * |
| **44** | **+9q21.11q34.3** | * |
| **45** | **+9q21.11q34.3** |  |
| **46** | **+9q21.11q34.3** | * |
| **47** | **+9q21.11q34.3** |  |
| **48** | **+9q21.11q34.3** | * |
| **49** | **+9q21.11q34.3** | * |
| **50** | **+9q21.11q34.3** | * |
| **51** | **+10p15.3q11.22** |  |
| **52** | **-11p15.5p12** | * |
| **53** | **-11q14.2q25** | * |
| **54** | **+11p15.5p11.12** | * |
| **55** | **+11q14.2q25** |  |
| **56** | **-12p13.33p12.3** | * |
| **57** | **+12q21.1q24.33** | * |
| **58** | **-13q22.2q34** |  |
| **59** | **+13q14.2q34** |  |
| **60** | **+19q11q13.43** |  |
| **61** | **-Xp11.4p11.21** | * |
| **62** | **-Xq21.32q28** | * |
| **63** | **+Xq22.1q26.3** |  |
| **64** | **-1q41q44** | * |
| **65** | **-1q42.12q44** | * |
| **66** | **+1q21.1q44** |  |
| **67** | **-2q11.1q37.3** | * |
| **68** | **-2q35q37.3** | * |
| **69** | **+2p25.3p24.1** | * |
| **70** | **+3q22.3q29** |  |
| **71** | **-4q34.1q35.2** | * |
| **72** | **-4q32.1q35.2** | * |
| **73** | **+4q24q35.2** | * |
| **74** | **+4q24q35.2** |  |
| **75** | **-5p15.33p13.2** | * |
| **76** | **-7q21.12q36.3** | * |
| **77** | **-8q21.13q24.3** | * |
| **78** | **-9p24.3p12** |  |
| **79** | **+9q21.11q34.3** | * |
| **80** | **+9q21.11q34.3** | * |
| **81** | **-10p15.3p11.1** | * |
| **82** | **-11p15.5p11.12** |  |
| **83** | **+11q11q25** | * |
| **84** | **-12p13.33p11.1** | * |
| **85** | **+12p13.33p11.1** | * |
| **86** | **+14q24.3q32.33** | * |
| **87** | **-1q21.2q44** |  |
| **88** | **+1q21.1q44** | * |
| **89** | **+1q21.1q44** |  |
| **90** | **-2q14.3q33.1** |  |
| **91** | **-3q25.2q29** | * |
| **92** | **-3q24q29** |  |
| **93** | **+3p26.3p11.1** | * |
| **94** | **+3p26.3p25.1** |  |
| **95** | **-4p16.3p11** | * |
| **96** | **+5p15.33q14.3** |  |
| **97** | **+6q14.1q27** |  |
| **98** | **-7q31.31q36.3** | * |
| **99** | **+7q22.1q36.3** | * |
| **100** | **+7q11.21q36.3** |  |
| **101** | **+7q11.21q36.3** | * |
| **102** | **+7q21.2q36.3** |  |
| **103** | **-9p24.3p12** |  |
| **104** | **-9p24.3p21.3** | * |
| **105** | **+9p24.3p13.1** | * |
| **106** | **+9p24.3p13.1** | * |
| **107** | **-10q24.1q26.3** |  |
| **108** | **+10p15.3p11.1** | * |
| **109** | **-11q13.3q25** |  |
| **110** | **+11p15.5p11.12** |  |
| **111** | **-7p15.5p13** | * |
| **112** | **-12p13.33p11.1** | * |
| **113** | **+12q11q24.33** | * |
| **114** | **-13q21.1q34** | * |
| **115** | **-16q11.2q24.3** | * |
| **116** | **+16p13.3p11.2** | * |
| **117** | **+16q11.2q24.3** | * |
| **118** | **+16q21q24.3** | * |
| **119** | **+16q11.2q24.3** | * |
| **120** | **-17p13.3p11.2** | * |
| **121** | **-20p13p11.1** | * |
| **122** | **-1p36.33p13.2** | * |
| **123** | **-1q23.1q44** | * |
| **124** | **-2p25.3p15** | * |
| **125** | **-2p25.3p16.3** |  |
| **126** | **-2q31.1q34** |  |
| **127** | **-2q33.1q37.3** | * |
| **128** | **-2q13q37.3** | * |
| **129** | **+2q32.2q37.3** |  |
| **130** | **+2q33.1q37.3** |  |
| **131** | **-3q26.2q29** |  |
| **132** | **-3q25.2q29** | * |
| **133** | **+3p26.3p11.1** | * |
| **134** | **+3p26.3p11.1** |  |
| **135** | **-4q25q35.2** | * |
| **136** | **-4q31.3q35.2** | * |
| **137** | **+4p16.3p11** | * |
| **138** | **+4q32.2q35.2** |  |
| **139** | **-5q12.3q35.3** |  |
| **140** | **-5q11.1q35.3** |  |
| **141** | **-5q11.1q35.3** | * |
| **142** | **+5q31.1q35.3** |  |
| **143** | **-6p25.3p22.3** | * |
| **144** | **-6p22.3p15.3** | * |
| **145** | **-6q13q27** | * |
| **146** | **+6p25.3q16.1** |  |
| **147** | **+6q22.31q27** | * |
| **148** | **-7p22.3p11.1** | * |
| **149** | **-7q21.11q36.3** |  |
| **150** | **+7p22.3p11.1** |  |
| **151** | **+9p24.3p21.1** | * |
| **152** | **+9q21.11q34.3** |  |
| **153** | **-10p11.23p15.3** | * |
| **154** | **-10q24.32q26.3** | * |
| **155** | **-10q11.21q26.3** | * |
| **156** | **-11p15.5p14.3** | * |
| **157** | **-11p15.5p11.2** | * |
| **158** | **+11p15.5p11.12** |  |
| **159** | **+13q31.1q34** | * |
| **160** | **+13q31.1q34** | * |
| **161** | **-14q23.3q32.33** | * |
| **162** | **-14q31.3q32.33** | * |
| **163** | **-15q24.3q26.3** | * |
| **164** | **-16p13.3p13.12** | * |
| **165** | **-16q11.2q24.3** |  |
| **166** | **+16p13.3p11.1** | * |
| **167** | **-17q21.31q25.3** | * |
| **168** | **+18p11.32p11.1** | * |
| **169** | **+20p13p12.1** |  |
| **170** | **+20q11.21q13.33** |  |
| **171** | **-7q24q28** |  |
| **172** | **+3p26.3p25.2** | * |
| **173** | **+3q11.1q25.1** | * |
| **174** | **+4q28.3q35.2** | * |
| **175** | **-5q11.2q35.3** | * |
| **176** | **-10q23.33q26.3** | * |
| **177** | **-14q21.2q32.33** |  |
| **178** | **+7p22.3p15.2** |  |
| **179** | **-13q13.1q21.33** | * |
| **180** | **-1q41q44** | * |
| **181** | **+11q11q25** |  |
| **182** | **-7p22.3p11.1** |  |
| **183** | **+20p13p11.1** |  |
| **184** | **-1p36.33p32.1** |  |
| **185** | **-1p36.33p31.3** |  |
| **186** | **-1q21.2q44** | * |
| **187** | **+1q21.1q44** |  |
| **188** | **+1q21.1q44** | * |
| **189** | **-2p25.3p13.1** |  |
| **190** | **-2p25.3p22.1** |  |
| **191** | **-2p11p25.1** | * |
| **192** | **-2q21.1q37.3** |  |
| **193** | **-2q11.1q37.3** | * |
| **194** | **+2q33.1q37.3** | * |
| **195** | **-4q31.23q35.2** | * |
| **196** | **+4p15.2p16.3** | * |
| **197** | **+4q28.3q35.2** | * |
| **198** | **+4q11q35.2** | * |
| **199** | **+5q31.2q35.3** | * |
| **200** | **+5q23.3q35.3** | * |
| **201** | **+5q11.1q35.3** | * |
| **202** | **-6p25.3p22.3** | * |
| **203** | **+6q15q27** |  |
| **204** | **+6q13q27** | * |
| **205** | **+6q16.1q27** |  |
| **206** | **+6q14.3q27** |  |
| **207** | **-7q33q36.3** |  |
| **208** | **+7q22.3q36.3** |  |
| **209** | **-8q23.3q24.3** | * |
| **210** | **-8q21.2q24.3** | * |
| **211** | **+8p23.3p11.1** |  |
| **212** | **+8q21.2q24.3** | * |
| **213** | **+8q11.1q24.3** |  |
| **214** | **+8q13.1q24.3** | * |
| **215** | **-9p24.3p22.1** |  |
| **216** | **-9q31.2q34.3** |  |
| **217** | **-11p12q25** |  |
| **218** | **+11p15.5p11.12** |  |
| **219** | **-12p13.33p11.1** |  |
| **220** | **+12p13.33q14.1** |  |
| **221** | **-15q22.2q26.3** |  |
| **222** | **+16q11.2q24.3** |  |
| **223** | **+17p13.3p12** |  |
| **224** | **-Xq11.1q28** | * |
| **225** | **-1p13.3p36** | * |
| **226** | **-1q32.1q44** | * |
| **227** | **+1q21.1q44** | * |
| **228** | **-2p25.3p13.3** | * |
| **229** | **-2p25.3p11.2** |  |
| **230** | **-2q11.1q37.3** |  |
| **231** | **-2q31.1q37.3** |  |
| **232** | **-4p16.3p14** |  |
| **233** | **-4q13.1q35.2** | * |
| **234** | **-4q32.3q35.2** | * |
| **235** | **-5q21.2q35.3** | * |
| **236** | **+5q11.1q35.3** | * |
| **237** | **+6q23.3q27** | * |
| **238** | **+6q16.3q27** | * |
| **239** | **+7q21.2q36.3** | * |
| **240** | **+8q23.1q24.3** |  |
| **241** | **-9q21.11q34.3** | * |
| **242** | **+9q21.11q34.3** |  |
| **243** | **-10p15.3p12.2** |  |
| **244** | **-10q22.3q26.3** | * |
| **245** | **+11q14.3q25** |  |
| **246** | **-12q21.31q24.33** | * |
| **247** | **+12q22q24.33** | * |
| **248** | **+12q23.1q24.33** |  |
| **249** | **-17q22q25.3** |  |
| **250** | **+18p11.32p11.1** |  |
| **251** | **-20p13p11.1** |  |
| **252** | **-1q21.1q44** |  |
| **253** | **+1q21.1q44** | * |
| **254** | **-2q21.1q37.3** | * |
| **255** | **+2q13q37.3** |  |
| **256** | **-5p15.33p13.2** | * |
| **257** | **-7p22.3p11.1** | * |
| **258** | **+8q21.3q24.3** | * |
| **259** | **+6q21.11q34.3** | * |
| **260** | **+9q21.11q34.3** | * |
| **261** | **-10q11.23q26.3** | * |
| **262** | **+10q11.22q26.3** |  |
| **263** | **-11p15.5p11.12** |  |
| **264** | **+11p15.5p11.12** |  |
| **265** | **+11q14.3q25** | * |
| **266** | **-2q12.1q37.3** |  |
| **267** | **+2p25.3p22.2** | * |
| **268** | **-4q28.3q35.2** | * |
| **269** | **-5p15.33p11** | * |
| **270** | **-7q31.1q36.3** |  |
| **271** | **-7q32.1q36.3** | * |
| **272** | **-9p24.3p21.2** | * |
| **273** | **+11q23.3q25** | * |
| **274** | **-7p22.3q22.1** |  |
| **275** | **-6p25.3p21.31** |  |
| **276** | **-6q14q27** |  |
| **277** | **+1q32.1q44** |  |
| **278** | **+5q21.3q35.3** |  |
| **279** | **-18q12.1q23** |  |
| **280** | **-1q25.3q44** |  |
| **281** | **-5q23.1q35.3** |  |
| **282** | **-4p16.3p16.1** |  |
| **283** | **+1q32.1q44** |  |
| **284** | **+Xp22.33p21.3** |  |
| **285** | **-6q13q27** |  |
| **286** | **-4q34.2q35.2** |  |
| **287** | **+9q21.11q34.3** |  |
| **288** | **-5q14.1q35.3** |  |
| **289** | **+9p24.3p13.1** |  |
| **290** | **+3p26.3p22.3** |  |
| **291** | **+1p36.33p31.1** |  |
| **292** | **+11q13.3q25** |  |
| **293** | **-6q26q27** |  |
| **294** | **+6q21q25.3** |  |
| **295** | **-1p36.33p35.3** |  |
| **296** | **-6q11.1q27** | * |
